# One session of high-intensity interval training (HIIT) every 5 days, improves muscle power but not static balance in lifelong sedentary ageing men: A randomized controlled trial

**DOI:** 10.1097/MD.0000000000006040

**Published:** 2017-02-10

**Authors:** Nicholas F. Sculthorpe, Peter Herbert, Fergal Grace

**Affiliations:** a Institute of Clinical Exercise & Health Sciences, School of Science and Sport, University of the West of Scotland, Hamilton, South Lanarkshire, Scotland, UK; b University of Wales Trinity Saint David, Camarthen Campus, Camarthen, Wales, UK; c Faculty of Health, Federation University Australia, Mt Helen Campus, Mt Helen, Victoria, Australia.

**Keywords:** ageing, dynapenia, low-frequency high-intensity interval training, muscle quality, sarcopenia

## Abstract

**Background:**

Declining muscle power during advancing age predicts falls and loss of independence. High-intensity interval training (HIIT) may improve muscle power, but remains largely unstudied in ageing participants.

**Methods:**

This randomized controlled trial (RCT) investigated the efficacy of a low-frequency HIIT (L_*f*_HIIT) intervention on peak muscle power (peak power output [PPO]), body composition, and balance in lifelong sedentary but otherwise healthy males.

**Methods:**

Thirty-three lifelong sedentary ageing men were randomly assigned to either intervention (INT; n = 22, age 62.3 ± 4.1 years) or control (n = 11, age 61.6 ± 5.0 years) who were both assessed at 3 distinct measurement points (phase A), after 6 weeks of conditioning exercise (phase B), and after 6 weeks of HIIT once every 5 days in INT (phase C), where control remained inactive throughout the study.

**Results:**

Static balance remained unaffected, and both absolute and relative PPO were not different between groups at phases A or B, but increased significantly in INT after L_*f*_HIIT (*P* < 0.01). Lean body mass displayed a significant interaction (*P* < 0.01) due to an increase in INT between phases B and C (*P* < 0.05).

**Conclusions:**

6 weeks of L_*f*_HIIT exercise feasible and effective method to induce clinically relevant improvements in absolute and relative PPO, but does not improve static balance in sedentary ageing men.

## 1 Introduction

During advancing age, muscle power, the product of muscle force and contraction velocity, declines earlier and more precipitously than muscle strength.^[1]^ Consequently, muscle power is the more discriminant predictor of future falls and loss of independence in older adults.^[2]^ The term “dynapenia” has emerged to describe this phenomenon,^[3]^ and together with the more well-known sarcopenia, better describes the totality of change to ageing muscle.

Exercise per se, is a well-established moderator of the age-associated decline in muscle power.^[2]^ However, the most effective and feasible exercise prescription to ameliorate age-associated dynapenia, remains to be determined. To date, preventative gerontological studies have primarily focused on resistance-based exercises. Notably, a systematic review and meta-analysis by Gillespie et al^[4]^ identify that participation by older adults in exercise programs reduces the incidence of serious falls. Similarly, the viability of high-intensity interval training as a method of maintaining muscular power during advancing age has been largely neglected. This is despite the emerging evidence that high-velocity exercise movements have the additional benefit of improving balance in ageing cohorts.^[5]^

Since the landmark study of Åstrand etal^[6]^ in 1960 in athletes, high-intensity interval training (HIIT) has become an accepted exercise modality to improve aerobic fitness which is enjoying a recent re-emergence as a practicable method to improve cardiovascular health amongst a variety of young and adult populations. HIIT is characterized by brief, intermittent bursts of vigorous exercise, interspersed by periods of rest or low-intensity recovery.^[7]^ There is an emergent body of evidence that endorses HIIT as an effective alternative to traditional endurance training that can yield improvements in both cardio-respiratory fitness and variety of health outcomes. However, HIIT remains largely unexplored in otherwise healthy sedentary older adults,^[8]^ encouraging a recent call for such studies in ageing cohorts.^[9]^

Traditionally, HIIT programs include several short efforts of high-intensity work performed each session, and with sessions performed thrice weekly. However, our research group have recently demonstrated that older men take longer to recover from a single session of HIIT than younger counterparts,^[10]^ with men aged 60 years requiring 5 days to recover peak power output (PPO), compared with men aged 25 years requiring 3 days. This implies that standard HIIT protocols should be adapted for exercise prescription in older adults. Consequently, HIIT might effectively be achieved in older adults by reducing the frequency of weekly sessions. Reducing training volume in this manner may be a more suitable alternative to improve muscle power during sedentary (but otherwise healthy) ageing. We hereafter term this exercise low-frequency HIIT (L_*f*_HIIT), to distinguish this form of high-intensity intermittent exercise from the traditional 3-weekly sessions.

We have recently described the potential for HIIT to improve muscle power in ageing men.^[11]^ Therefore, the purpose of this research article is to extend this previous work and to respond to the call in the literature^[9]^ by investigating the effectiveness of a L_*f*_HIIT program on components of leg power and static balance in otherwise healthy sedentary ageing males. We hypothesized that L_*f*_HIIT, subsequent to conditioning exercise, would produce clinically relevant improvements in leg power in otherwise healthy sedentary ageing compared with age-matched sedentary male controls. We further hypothesized that L_*f*_HIIT would improve balance in the group receiving the intervention.

## 2 Methods

### 2.1 Ethical approval

**Figure 1:**
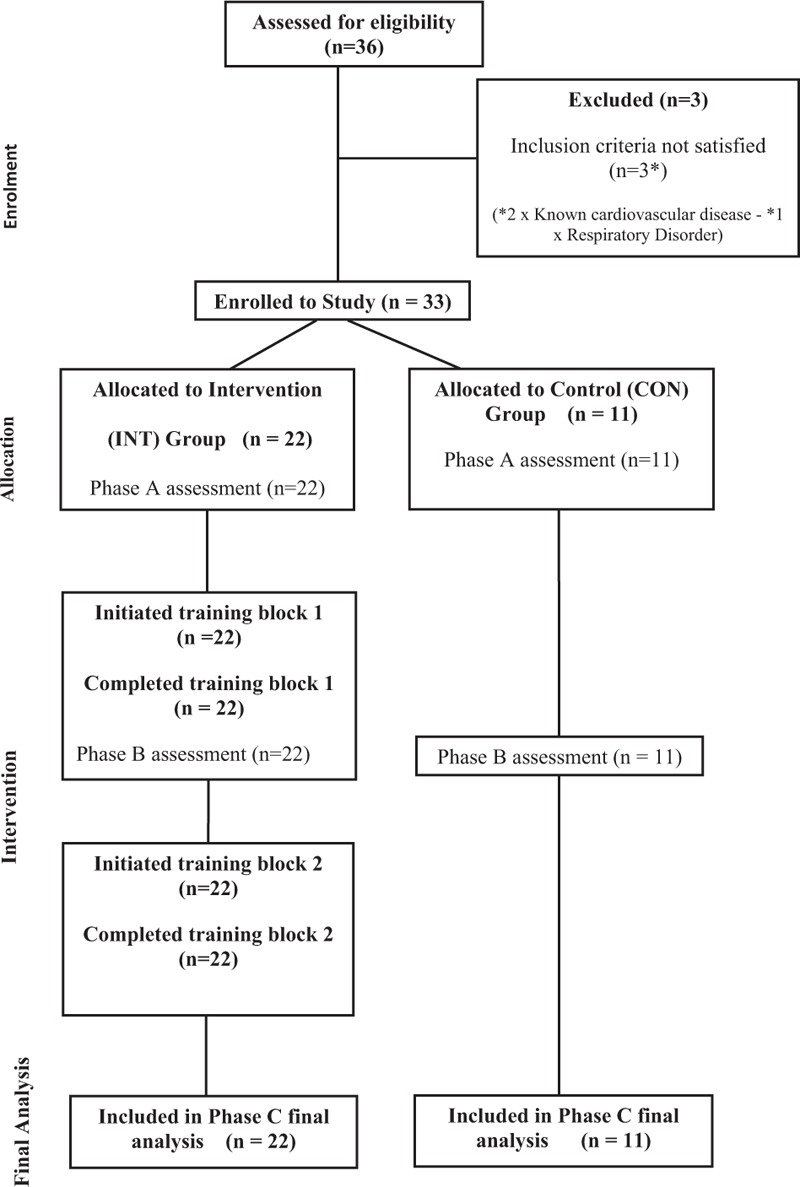
Flow diagram of participants randomized to either intervention (INT) or control (CON) through the 3 measurement phases of the study.

Participants consisted of sedentary male volunteers (n = 36) between the ages of 56 and 65 years. Inclusion criteria were that participants had not participated in any formal exercise training for a minimum of 30 years; all participants self-reported as not being involved in any regular physical activity for either recreational or work-related purposes. General medical practitioners (GPs) for each potential participant were required to provide a written letter of approval for their participant to enroll to the study. Participants were withdrawn if, in the opinion of their GP, they were not suitable to take part in strenuous physical activity. Three participants were subsequently withdrawn due to existing cardiovascular disease (n *=* 2) and respiratory disorder (n *=* 1). The flow of participants through the study is depicted in Fig. 1. The remaining participants completed a physical activity readiness questionnaire (PAR-Q) and provided written informed consent to participate in the study, which was approved by the University of the West of Scotland research ethics committee. After enrolment, cardiorespiratory fitness was established in an exercise physiology laboratory by indirect calorimetry using a ramp protocol on a cycle ergometer to exhaustion as described in greater detail elsewhere,^[12]^ where individual maximal heart rates were used to establish and confirm exercise intensity (heart rate reserve) during the study. Subsequently, participants (n *=* 33) were randomly assigned, in a 2:1 ratio, into either an intervention group (INT; n = 22) or a control group (CON; n = 11). Asymmetric randomization was considered appropriate due to the potential for high attrition rates in the intervention group resulting in underpowered statistical outcomes.^[13]^ Participant characteristics are outlined in Table 1.

**Table 1:**
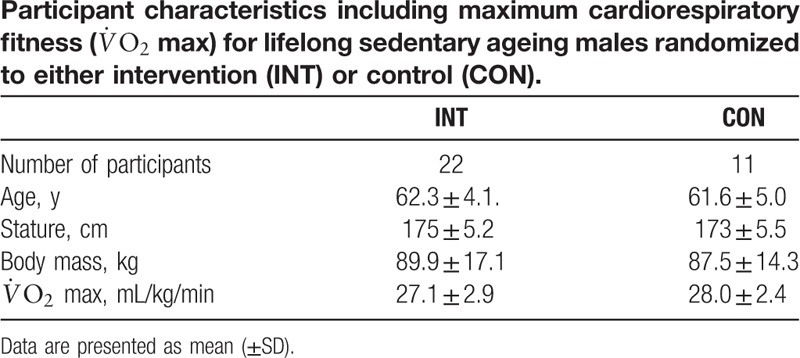


**Figure 2:**
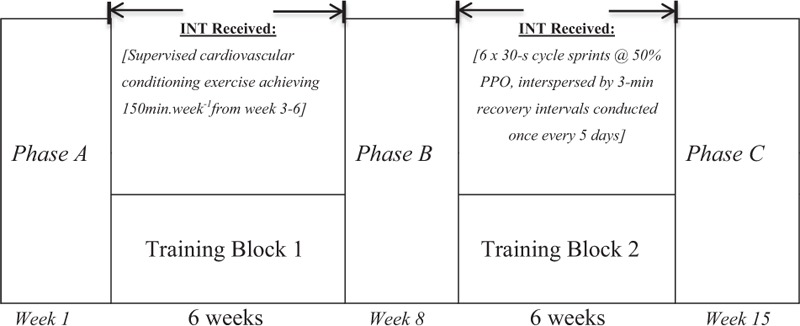
Schematic depicting study design incorporating 3 testing phases (A, B, and C) of 2 distinct training blocks for those receiving intervention (INT) and age-matched controls (CON) who remained inactive for duration of the study.

Due to the unknown effect of high-intensity exercise in sedentary older men, and to account for the effects of conditioning exercise, the study required 3 distinct measurement phases (A, B, and C) separated by 2 training blocks (1 and 2). Therefore, following randomization, INT undertook 6 weeks of supervised “preconditioning” exercise (training block 1), before undertaking a program of L_*f*_HIIT (training block 2), whereas CON remained inactive for the duration of the study. Participants were required to complete a minimum of 80% of training sessions to be included in the final data analysis. Figure 2 outlines a schematic of study design.

Calculation of sample power was based on previously published data regarding exercise-induced changes in Wingate PPO in younger cohorts after sprint interval training.^[14]^ This suggested an estimated effect size of 1.08, using an α of 0.05 and β of 0.8, and taking the asymmetric allocation into account resulted in a requirement of 23 participants in INT and 11 participants in CON. A priori power calculations were conducted using GPower V3.1, and 2:1 recruitment ratio was subsequently utilized to account for potential study accession within the intervention group.

### 2.2 Laboratory measures

On assessment Phases A, B, and C, participants arrived in the exercise physiology laboratory between the hours of 07.00 and 09.00 am, after an overnight fast and having abstained from strenuous physical activity for a minimum of 5 days. Participants were reminded in the information sheet to maintain standardized conditions before each assessment point, which included arriving in a hydrated state (500 mL water 30–60 min before assessment), having abstained from caffeine and alcohol consumption for 36 hours.

### 2.3 Body composition

Since body composition is a known covariate of increasing PPO, it is important to assess such changes. Therefore, body composition was determined using standard methods and described elsewhere.^[15]^ Body fat assessments were performed with participants hydrated and wearing minimal clothing. Participants stood on a multifrequency bioelectrical impedance device (Tanita MC180MA, Tanita Corp, Tokyo, Japan), ensuring bare feet were in contact with electrodes on the foot plate. This assessed body mass and relative proportions of fat and lean tissue, which were assessed over a 20-minute period, to account for technical variation. The coefficient of variation (CV) for the measurement of body composition total body mass (TBM), fat-free mass (FFM), and fat mass (FM) in our laboratory is <2%.

### 2.4 Determination of static balance

Static balance was assessed barefoot, using a Footscan portable foot pressure plate and stability software (RS Scan Labs Ltd, Ipswich, England, UK), which has previously been shown to be a valid and reliable tool to measure stability.^[16]^ The footplate measured the total distance and area covered by an individual's center of pressure over a 20-second interval. Static measures of balance were included, standing on both feet (*S*_B_) and standing balanced on the dominant foot (*S*_D_) (Table 2).

**Table 2:**
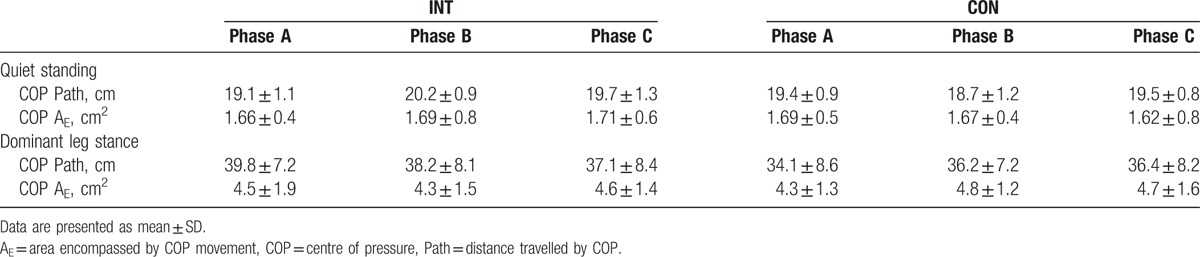
Centre of pressure measures for intervention (INT) and control (CON) participants, on enrolment to the study (phase A); after conditioning exercise (phase B), and after low-frequency, high-intensity exercise (L_*f*_HIIT; phase C).

### 2.5 Determination of peak power output

Both INT and CON participants were familiarized with the Herbert 6-second peak power test^[15]^ before commencing the study. This test was used to establish PPO at phases A, B, and C using a Wattbike Pro (Wattbike, Nottingham, UK) cycle ergometer employing a protocol detailed elsewhere.^[10]^ Briefly, each assessment of PPO was preceded with a 5-miuten warm-up on a Wattbike Pro set at a fixed resistance of (level 8, described as “heavy gearing”) and incorporated 2 brief cadence acceleration phases of approximately 3 seconds commencing after 90 and 180 seconds. The test employed a seated stationary start with dominant leg initiating the first down-stroke. The air braking resistance was set to level l0, and magnetic resistance set to level 1 (equating to 1045 W at 130 rpm and approximately 90–100 W increases for every further 5 rpm increase in cadence). After each cycle test, participants underwent 5 minutes of cool-down exercise on the Wattbike cycle, set at air brake resistance level 8. CV for the determination of PPO using this method in our laboratory is <1.6%, and we have previously demonstrated it to be a valid and reliable measure of PPO.^[10]^ PPO generated during phase B was used to establish the intensity of individual HIIT intervals during training block 2 (further detailed below).

### 2.6 Conditioning exercise (training block 1)

To prepare INT for the high-intensity exercise, this group underwent training block 1, which consisted of 6 weeks of personalized and supervised preconditioning exercise designed to progressively meet the American College of Sports Medicine (ACSM) guidelines^[17]^ of moderate-intensity cardiorespiratory exercise training consisting of ≥150 min per week (≥30 minutes/day on ≥5 days per week). This included with the familiarization of INT with Polar FT1 heart rate monitors (Polar Team System, Polar Electro Oy, Kempele, Finland), enabling the recording of exercise time, average and peak heart rate during exercise sessions. Progression of this conditioning exercise was achieved with systematic increments in weekly average heart rate reserve (HRR); week 1 to 2: 55% HRR; week 3 to 4: 60% HRR; week 5 to 6: 65% HRR, where the final 2 weeks incorporating short bursts of higher-intensity exercise into each session. Supervised exercise training modes provided options that included walking, walk/jogging, jogging, cycling, (flat terrain) cycling, (hill terrain), and adapted to suit the participants’ physical status and personal preference. Exercise compliance and heart rate data were recorded, and feedback on progress was administered to participants at the end of each exercise training session.

### 2.7 L_f_HIIT intervention (training block 2)

Training block 2 was based on a program of L_*f*_HIIT previously employed by our research group,^[8]^ which consisted of 1 L_f_HIIT session performed once every 5 days for 6 weeks (9 sessions). These sessions consisted of 5 minutes of warm up followed by 6 × 30-second sprints at 50% peak power on a cycle ergometer (Wattbike Pro, Nottingham, England, UK), each interspersed with 3-minute intervals of active recovery, which we have previously demonstrated to be feasible high-intensity protocol to achieve >90% HRR in a similarly aged cohort.^[18]^ The first 3 (3/9) L_*f*_HIIT sessions were used to familiarize the participants to high-intensity exercise, working at 40% of each participants’ peak power established during the 6-second peak power test^[19]^ at phase B. Subsequent sessions (6/9) were conducted at 50% of predefined PPO. This was the only exercise performed during this training block and preceded the final measurement phase (phase C). At each measurement phase, data were obtained 5 days after the last exercise session.

### 2.8 Statistical analysis

Data were analyzed using SPSS version 20.0. Q-Q plots were employed to confirm normal distribution of data. Training effects were compared using a 2 × 3 mixed design analysis of variance (ANOVA), comprising 3 within-group condition (phases A, B, and C) and 2 between-group conditions (INT and CON). Where significant interaction effects were evident, data were further investigated using post hoc pair-wise comparisons to identify within-group and between-group simple effects using a Bonferroni correction. An alpha value of *P* ≤ 0.05 was used to indicate statistical significance. Data are presented as mean ± standard deviation (SD); differences between conditions are reported as mean difference and 95% confidence intervals (95% CIs).

## 3 Results

### 3.1 Study compliance and adverse events

No participants were lost to follow-up. Among the participants who enrolled to the study, 100% completed phases A, B, and C, and there were no adverse events recorded either during the conditioning exercise (training block 1) or during the L_*f*_HIIT (training block 2).

### 3.2 Peak power output

**Figure 3:**
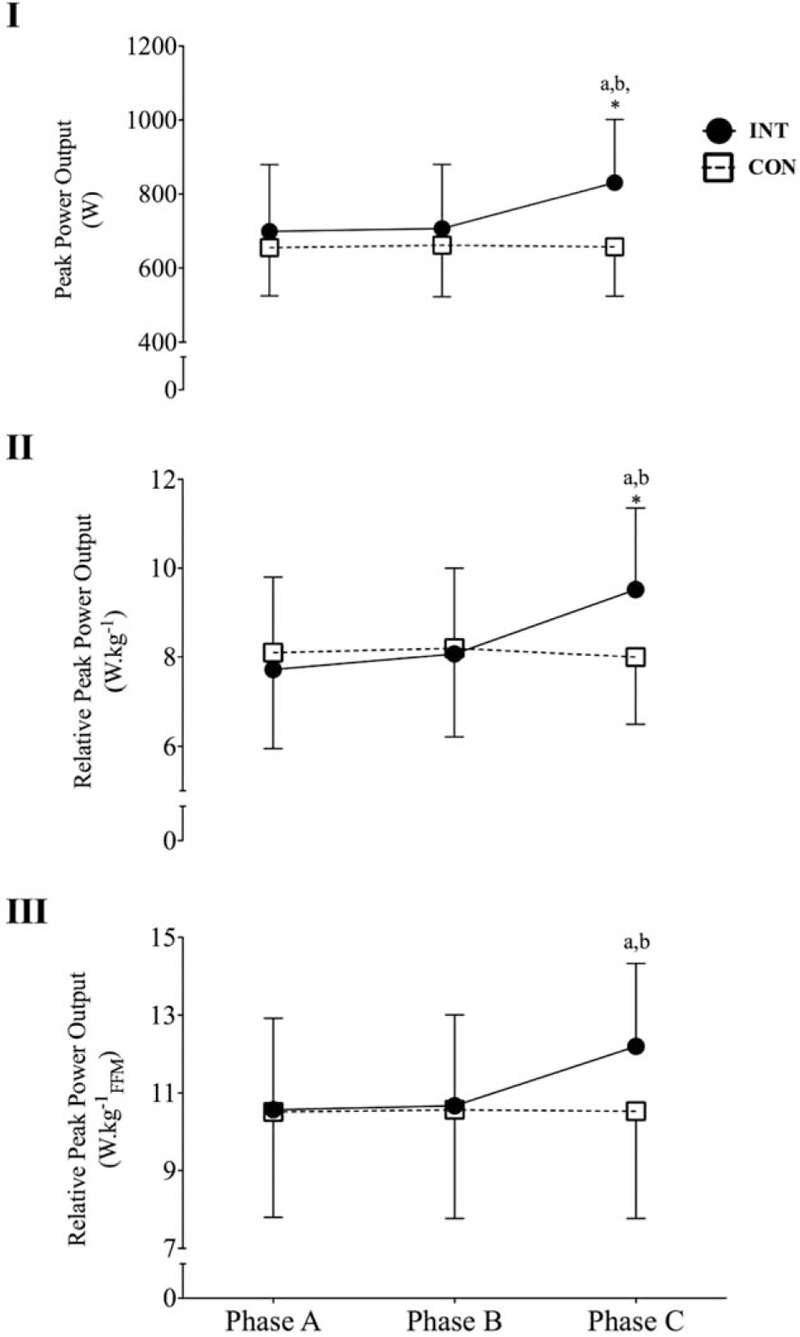
Changes in peak power output (I), peak power relative to total body mass (II), and peak power relative to fat-free mass (III) for the control group (CON) and the intervention group (INT) upon enrolment to the study (phase A), after 6 weeks of standard conditioning exercise (phase B) and after 6 weeks of L_*f*_HIIT (phase C). (∗) Indicates *P* < 0.05 versus CON at the same measurement phase; a: *P* < 0.01 versus phase A for the same group; b: *P* < 0.01 versus phase B for the same group.

For absolute peak power, there was a significant main effect of phase (*P* < 0.01), but not for group (*P* > 0.05); however, there was a significant interaction (*P* < 0.01). There were no differences between groups for absolute power at phase A (699.1 ± 180.1 vs 655.1 ± 130.1 W for INT and CON, respectively) or phase B (phase B = 706.5 ± 173.8 vs 661.8 ± 139 W for INT and CON, respectively). However, there were differences between groups at phase C (831.1 ± 170.6 vs 657.3 ± 133.1 W for INT and CON, respectively). INT participants did not increase absolute power between phases A and B (*P* > 0.05), but improved significantly between phases B and C (*P* < 0.01; 95% CI 90.2–159.0). PPO remained unchanged in CON at all measurement phases (*P* > 0.05 for all comparisons; Fig. 3I).

For rPPO (W/kg), there was a significant main effect of phase (*P* < 0.01), but not group (*P* > 0.05), and there was a significant interaction effect (*P* < 0.01). There were no between-group differences at phase A (7.71 ± 1.81 vs 8.1 ± 1.7 W/kg for INT and CON, respectively) or phase B (8.1 ± 1.7 vs 8.2 ± 1.8 W/kg for INT and CON, respectively), but at phase C, INT had significantly greater relative PPO than CON (9.5 ± 1.83 vs 8.0 ± 1.5 W/kg for INT and CON, respectively; *P* < 0.05). Within groups, INT demonstrated no increase between phases A and B (*P* > 0.05), but had a significant increase between phases B and C (*P* < 0.01; 95% CI 1.053–1.832), whereas there were no changes in relative PPO for CON (*P* > 0.05 for all comparisons; Fig. 3II*)*

The rPPO relative to FFM (rPPO_FFM_) was used to as a marker for changes in muscle quality. There was a main effect for phase (*P* < 0.01), but not for group (*P* *<* 0.05), and there was a significant interaction effect (*P* < 0.01). There were no differences between groups at phases A, B, or C (*P* > 0.05 for all comparisons). At phase C, there was a trend for a higher rPPO_FFM_ in INT (12.2 ± 2.1 vs 10.53 ± 2.76 W/kg_FFM_; *P* = 0.07, 95% CI −3.514 to 0.167). Within groups, INT did not change between phases A and B (*P* > 0.05); however, there was a significant increase in INT between phases B and C (10.67 ± 2.3 vs12.2 ± 2.1 W/kg_FFM_; *P* < 0.001, 95% CI 0.943–2.117). There were no changes in rPPO_FFM_ in CON (*P* > 0.05 for all comparisons; Fig. 3III).

### 3.3 Anthropometrics

**Figure 4:**
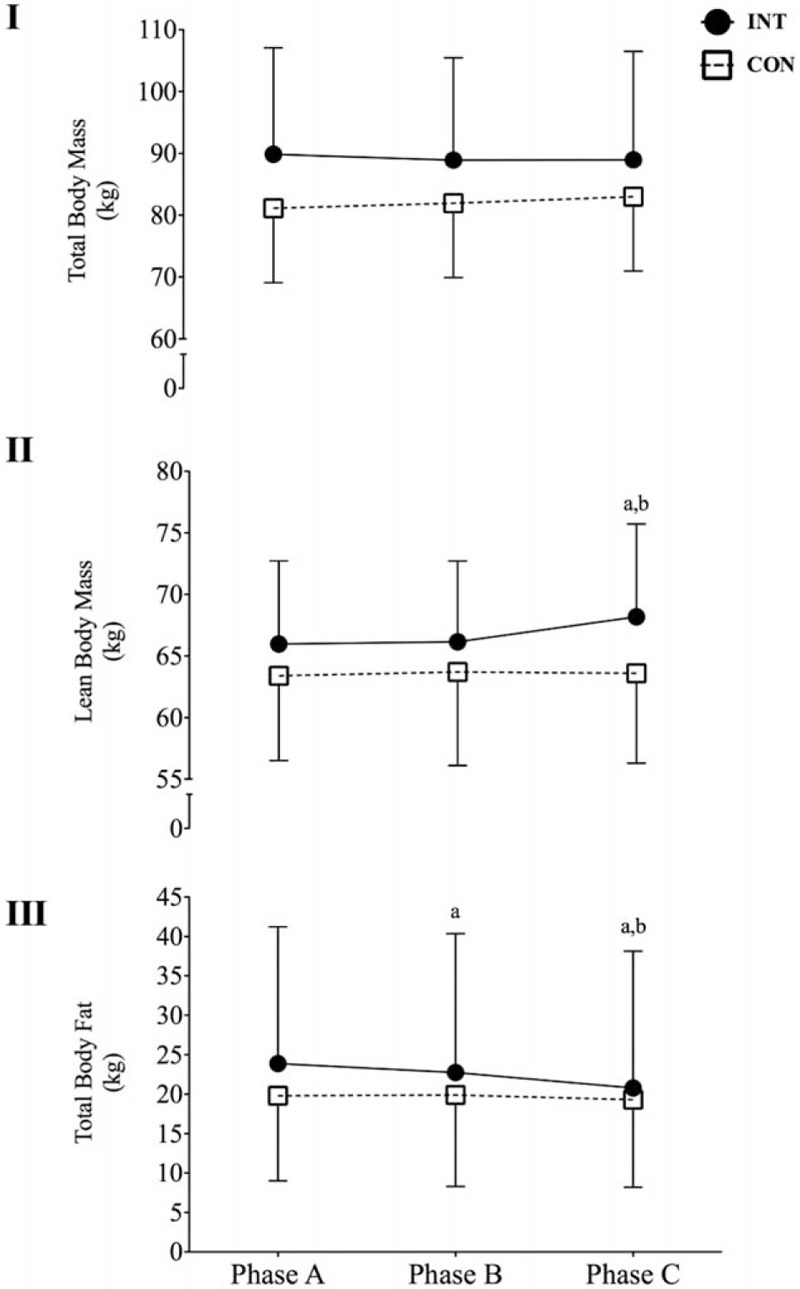
Changes in total body mass (I), lean body mass (II), and total body fat (III) for the control group (CON) and the intervention group (INT) upon enrolment to the study (phase A), after 6 weeks of standard conditioning exercise (phase B) and after 6 weeks of L_*f*_HIIT (phase C). a: *P* < 0.05 versus phase A for the same group; b: *P* < 0.05 versus phase B for the same group.

For TBM, there was no main effect for phase (*P* > 0.05), nor for group (*P* < 0.05), nor was there a significant interaction effect (*P* > 0.05). For lean body mass (LBM), there was a significant main effect for phase (*P* < 0.05), but not for group (*P* > 0.05), and a significant interaction between the 2 (*P* < 0.05). Between groups, there was no difference at any phase (*P* > 0.05 for all comparisons; phase A = 65.9 ± 6.7 kg vs 63.4 ± 6.9 kg; phase B = 66.1 ± 6.6 vs 63.7 ± 7.6 kg; phase C = 68.1 ± 7.5 vs 63.6 ± 7.3 kg for INT and CON, respectively) (Fig. 4I*)*. Within groups, LBM did not change in INT between phases A and B (*P* > 0.05), but there was a significant increase between phases B and C (*P* < 0.05, 95% CI 0.545–3.512), whereas CON did not experience any changes in LBM between any phases (*P* > 0.05 for all comparisons) (Fig. 4II*)*. Total body fat (TBF) demonstrated a significant main effect of phase (*P* < 0.01), but not group (*P* > 0.05), and a significant interaction between the phase and group (*P* < 0.05). Between groups, there were no significant differences at any phase (phase A = 23.9 ± 17.3 vs 19.8 ± 10.8 kg; phase B = 22.8 ± 17.6 vs 19.9 ± 11.6 kg; phase C = 20.8 ± 17.3 vs 19.3 ± 11.1 kg for INT and CON, respectively; *P* > 0.05 for all phases). Within groups, INT demonstrated significant decrease in TBF between phases A and B (*P* < 0.05, 95% CI 0.129–2.128), and between phases B and C (*P* < 0.05, 95% CI 0.392–3.551). CON experienced no change in TBF between any phase (*P* > 0.05 for all comparisons) (Fig. 4III).

### 3.4 Balance and flexibility

There was no main effect for phase (*P* > 0.05), group (*P* > 0.05), nor any interaction effects for any measures of static balance (Table 2).

## 4 Discussion

The main finding of the present study is that 6 weeks of L_*f*_HIIT, subsequent to conditioning exercise, is a feasible and effective method to improve PPO and rPPO in otherwise healthy sedentary ageing men. A further finding was that neither standard conditioning, nor L_*f*_HIIT resulted in any improvement in measures of static balance that were assessed in this study. These findings have important implications for exercise prescription in older populations.

### 4.1 Standard conditioning exercise

In the present study, general conditioning (phase A) had no effect on PPO or rPPO in INT. Previously, low to moderate intensity training has been shown to improve PPO calculated from vertical jump height in similarly aged females^[20]^; however, similar data in males are lacking. Indeed, the ACSM position statement^[17]^ relating to exercise for older individuals neglects to consider any effects of aerobic exercise training on measures of peak muscle power. Nevertheless, the present study failed to find any effect of moderate-intensity exercise on PPO in older males. This is likely attributable to the specificity of neuromuscular and metabolic adaptations of skeletal muscle in response to chronic exercise.

### 4.2 L_*f*_HIIT

The most comprehensive data currently available on low volume HIIT is a recently published meta-analysis by Weston et al.^[9]^ However, as indicated by the authors, their results deal exclusively with younger adult participants, as there are no previously published data regarding the effects of low-volume HIIT on indices of muscular power or body composition in healthy older participants. Indeed, Weston et al^[9]^ identify that of those studies reporting PPO, the oldest cohort previously studied had a mean age of 32 years.^[21]^ Correspondingly, this study represents the oldest cohort to undertake a HIIT program, to date. Weston et al^[9]^ report the effect of low-volume HIIT on PPO in younger cohorts as being unclear, reporting a small average increase and wide CIs (1.8% ± 5%), meaning that in some cases, there was a negative effect of low-volume HIIT training interventions. This was calculated using the difference between control and intervention groups, with the small effect partly due to PPO improvements in control groups. Using the same calculation, the present study reports an overall effect on PPO that is an order of magnitude greater (26.5%) after L_*f*_HIIT, when comparing INT with CON, and a within-group increase of 17% for INT between phases B and C.

One potential explanation for the greater effect in the present study relates to participants’ age. Since normal ageing is associated with a progressive decline in PPO,^[2]^ it is likely that participants in the present study had a low PPO at baseline, and consequently had a much larger scope for improvement than studies of younger participants. Although further comparison with available literature is hindered, as this is the first RCT study using older participants, it does provide the first evidence that L_*f*_HIIT may be an age-specific training method to increase PPO.

Similarly, there are other differences with previous investigations that merit discussion. In particular, the present study used a low-frequency and low-volume HIIT model as opposed to previous investigations that have utilized a low exercise volume per session, but have maintained the traditional frequency of 3 sessions per week. This is of particular importance as other variables within the present study are comparable with previous investigations using low-volume HIIT. These include variables such as intensity, effort duration, total effort per session, work-rest ratio, and number of efforts per session.^[9]^ Consequently, the large improvements in PPO and rPPO in INT in the present study are even more surprising, given the use of both similarly low session volumes, and reduced training frequency (once every 5 days giving a mean frequency of 1.4 sessions/wk), which was less than half that of previous investigations. Correspondingly, the improvements in INT were in response to a total of 27 minutes of HIIT exercise across the 6-week period.

We have previously reported that when compared with their younger counterparts, older participants require longer recovery periods after a single bout of HIIT,^[15]^ with PPO recovery occurring within 5 days. Consequently, it appears that in older participants, allowing full recovery between bouts may facilitate muscle adaptation to HIIT and should be considered when programming training or L_*f*_HIIT protocols for older participants. It is evident from the present study that reducing exercise frequency in older cohorts is not detrimental to improvements in muscle power. It seems likely that the extended recovery between bouts allows for more complete recovery and adaptation (a process that may be slower in older individuals), and can prevent accumulated fatigue that might occur should training sessions be programmed too frequently.

The vast majority of studies investigating muscle power in ageing participants have used resistance exercise, high-speed power movements (normally adapted from traditional resistance exercises), or a combination thereof. Whereas high-velocity movements are widely considered to be more effective than high-load, low-velocity resistance exercise for improving muscle power,^[5]^ comparisons with the present study are difficult since the outcome measures used in individual studies are specific to their training intervention, with the consequence that the utility of comparing relative efficacy becomes questionable. For example, Sayers and Gibson^[22]^ report an approximate 3.5-fold improvement over control subjects in PPO generated against a resistance of 40% of participants leg press 1 repetition maximum (1RM). Given the mechanical and metabolic differences between a single expression of power during a leg press versus 30 seconds of high-intensity cycling, it is difficult to conclude the superiority of one intervention over another.

### 4.3 Anthropometrics

During initial conditioning, INT underwent a small reduction in body fat in the absence of any change to LBM. However, after L_*f*_HIIT, the reduction in body fat accelerated with a concomitant increase in LBM. Increased daily energy expenditure during general conditioning is the most likely explanation for the small reduction in body fat during that phase. The effectiveness of moderate-intensity exercise to reduce body fat in sedentary individuals has been previously reported^[17]^ and further substantiated by the present data. The mechanism behind a reduction in body fat after L_*f*_HIIT is unclear. As outlined above, the total high-intensity exercise time during that phase of the study was 27 minutes over 6 weeks (this increases to a total of 4.5 hours of exercise when recovery, warm-up, and cool-down periods were included). It would appear unlikely that reductions in body fat were mediated by increased calorie expenditure. One unexplored avenue is that HIIT participants may self-select higher levels of physical activity out-with the HIIT intervention which has been demonstrated in adolescents,^[23]^ but remains to be examined in adult and ageing populations. There are numerous reports of HIIT training resulting in reductions in TBM,^[24]^ TBF,^[25]^ and total visceral adipose tissue.^[26]^ The mechanisms underpinning reductions in body fat after HIIT are poorly understood, but have been postulated as being related to improved exertional, postexercise, and resting fat oxidation,^[26]^ and/or appetite suppression.^[27]^ The relative change in TBF in the present study (8.8% between phase B and phase C) is similar to changes reported in other HIIT studies, utilizing greater frequencies than is the case here.^[24,25]^ The present study extends these results in 2 ways. First, by demonstrating that the effect is similarly evident in previously sedentary, but otherwise healthy ageing men. And secondly, by demonstrating that the effect persists despite low weekly exercise frequency and low total exercise volume.

In contrast to its effects on fat mass, the effect of HIIT on lean body mass has not been extensively investigated. Combined endurance and HIIT training has previously been shown to increase thigh cross-sectional area over 16 weeks in middle-aged diabetic men. More recently, Gillen et al^[28,29]^ demonstrated increases in FFM in the leg and gynoid regions of overweight young females after 6 weeks of low-volume HIIT performed 3 times per week. In the present study, muscle hypertrophy was not directly measured, but clearly cannot be ruled out as a contributor to increased LBM. Similarly, although not measured in this study, exercise-induced changes to male sex hormones may have indirectly influenced PPO through interactions with muscle hypertrophy/function.^[30]^ However, the tenet for this phenomenon is not well described in the ageing male literature,^[31,32]^ and the latest evidence introduces the potential for a significant role of insulin-like growth factor in the exercise response in ageing men.^[33]^ Nonetheless, increases in muscle mass in this study would have the additional benefit of increasing resting metabolic rate, which may also explain the accelerated reduction in TBF after L_*f*_HIIT. Nevertheless, whatever the basis for increased LBM, the present study builds on previous work by demonstrating that in older cohorts, increases in LBM are possible, and can occur despite low exercise frequency and volumes.

In addition to adverse changes in muscle mass and body composition, ageing is also associated with decrements in muscle quality (defined as force production per unit of muscle).^[34]^ Although not investigated directly in the present study, PPO relative to FFM (rPPO_FFM_) may be used as a surrogate, though imperfect measure of muscle quality. In the present study, L_*f*_HIIT resulted in significant increases in rPPO_FFM_ in INT. This suggests that along with the increases in FFM, L_*f*_HIIT may also improve muscle quality. These changes, when viewed alongside with the increases in PPO, the small decreases in TBF, the low time requirements, and the high levels of adherence, suggests L_*f*_HIIT may be a potent tool for improving measures predictive of future frailty in older sedentary populations. Our use of rPPO_FFM_ as a surrogate marker for muscle quality is not without significant limitations, though our findings suggest that this is an avenue for further study. As such, the 56–65 yrs cohort in this study are good candidates for high intensity training. Whereas undoubtedly aged, this demographic, frequently termed “the young old” typically have many years of minimally diminished functional capacity remaining and are less likely to have a mobility limiting disorders. In the absence of epidemiological evidence for HIIT to improve “lifespan,” or indeed, long-term health outcomes in previously sedentary ageing cohorts, this study provides preliminary evidence for L_*f*_HIIT to induce short-term improvements in muscle power. With careful exercise prescription, L_*f*_HIIT-induced improvements in muscle power could potentially be carried through into older age, thus positively impacting the exercisers’ “health-span.” With this in mind, replication of the present findings and longer-term follow-up studies in male and female cohorts are strongly encouraged.

Neither conditioning exercise nor L_*f*_HIIT was effective at improving indices of balance in INT. Previous investigations have reported that power training can improve balance in older participants.^[1,35]^ The reasons for the lack of effect of either training regimen in the present study may be due to a number of factors. Most previous studies have used functional movements using weighted vests^[35]^ or high-velocity movements adapted from traditional resistance training exercises.^[1]^ In contrast, stationary cycle ergometry includes 5 points of contact with a stable ergometer, and thus requires less corrective stability than other forms of high-velocity exercise. It is possible that stationary cycle ergometry is sufficiently stable and effectively limits improvements in balance. Alternatively, others have reported no detrimental effects on balance in older participants who have maintained moderate physical activity (regular walking) after retirement.^[36]^ Given that the age and static balance results of the present cohort were similar to those reported by Melzer et al, it is equally possible that our participants had no significant balance impairment, and thus any training effects may be minimal. In addition, it may be that the static balance test utilized in the study was insufficiently challenging to identify improvements after the training program. Consequently, future work in similar populations may wish to employ more challenging assessments of participants balance.

### 4.4 Study limitations

The present study has some important limitations that should be noted. One concerns the proximity (11 days) of the conditioning exercise (training block 1) to the L_*f*_HIIT intervention (training block 2), which makes it impossible to rule out the contribution of conditioning exercise to the overall effect on INT after L_*f*_HIIT. However, given the randomized controlled design and the lack of difference between groups at phase B, the difference in PPO evident at phase C is almost certainly due to the L_*f*_HIIT intervention. As the effects of high-intensity exercise are unstudied in older cohorts, the inclusion of the conditioning exercise during phase A was deemed a prudent means of gradually introducing the previously inactive INT cohort to the rigors of high-intensity training. This decision appears justified as there was complete adherence to the L_*f*_HIIT after conditioning. Similarly, the ACSM has recently indicated that conditioning training should be employed before undertaking high-intensity interval training.^[37]^

In conclusion, the novel findings of the present study are that L_*f*_HIIT is both feasible and effective as a training modality to increase lower limb muscle power in sedentary ageing men. However, when performed using stationary cycle ergometry, it does not improve balance in otherwise healthy sedentary ageing males. This study provides strong supporting evidence for the inclusion of L_*f*_HIIT when prescribing exercise to improve lower-limb power in ageing cohorts.

## References

[1] Orr R de Vos NJ Singh NA Power training improves balance in healthy older adults. J Gerontol Ser A Biol Sci Med Sci 2006;61:78–85. 16456197 10.1093/gerona/61.1.78

[2] Reid KF Fielding RA Skeletal muscle power: a critical determinant of physical functioning in older adults. Exercise Sport Sci Rev 2012;40:4–12. 10.1097/JES.0b013e31823b5f13 PMC3245773 22016147

[3] Clark BC Manini TM Sarcopenia=/=dynapenia. J Gerontol Ser A Biol Sci Med Sci 2008;63:829–34. 18772470 10.1093/gerona/63.8.829

[4] Gillespie LD Robertson MC Gillespie WJ Interventions for preventing falls in older people living in the community. Cochrane Database Syst Rev 2012;9:CD007146. 10.1002/14651858.CD007146.pub3 PMC8095069 22972103

[5] Tschopp M Sattelmayer MK Hilfiker R Is power training or conventional resistance training better for function in elderly persons? A meta-analysis. Age Ageing 2011;40:549–56. 21383023 10.1093/ageing/afr005

[6] Åstrand I Åstrand PO Christensen EH Intermittent muscular work. Acta Physiol Scand 1960;48:448–53. 13794890 10.1111/j.1748-1716.1960.tb01879.x

[7] Gibala MJ Little JP Macdonald MJ Physiological adaptations to low-volume, high-intensity interval training in health and disease. J Physiol 2012;590 (Pt 5):1077–84. 22289907 10.1113/jphysiol.2011.224725 PMC3381816

[8] Knowles AM Herbert P Easton C Impact of low-volume, high-intensity interval training on maximal aerobic capacity, health-related quality of life and motivation to exercise in ageing men. Age (Dordrecht, Netherlands) 2015;37:25. 10.1007/s11357-015-9763-3 PMC4359174 25773069

[9] Weston M Taylor KL Batterham AM Effects of low-volume high-intensity interval training (HIT) on fitness in adults: a meta-analysis of controlled and non-controlled trials. Sports Med 2014;44:1005–17. 24743927 10.1007/s40279-014-0180-z PMC4072920

[10] Herbert P Grace FM Sculthorpe NF Exercising caution: prolonged recovery from a single session of high-intensity interval training in older men. J Am Geriatr Soc 2015;63:817–8. 25900496 10.1111/jgs.13365

[11] Sculthorpe N Herbert P Grace FM Low-frequency high-intensity interval training is an effective method to improve muscle power in lifelong sedentary aging men: a randomized controlled trial. J Am Geriatr Soc 2015;63:2412–3. 26603065 10.1111/jgs.13863

[12] Grace FM Herbert P Ratcliffe JW Age related vascular endothelial function following lifelong sedentariness: positive impact of cardiovascular conditioning without further improvement following low frequency high intensity interval training. Physiol Rep 2015;3: pii: e12234. doi: 10.14814/phy2.12234. 10.14814/phy2.12234 PMC4387763 25626864

[13] Dumville JC Hahn S Miles JN The use of unequal randomisation ratios in clinical trials: a review. Contemp Clin Trials 2006;27:1–2. 16236557 10.1016/j.cct.2005.08.003

[14] Burgomaster KA Hughes SC Heigenhauser GJ Six sessions of sprint interval training increases muscle oxidative potential and cycle endurance capacity in humans. J Appl Physiol 2005;98:1985–90. 15705728 10.1152/japplphysiol.01095.2004

[15] Hayes LD Sculthorpe N Herbert P Six weeks of conditioning exercise increases total, but not free testosterone in lifelong sedentary aging men. Aging Male 2015;0:1–6. 10.3109/13685538.2015.1046123 26030347

[16] Gurney JK Kersting UG Rosenbaum D Between-day reliability of repeated plantar pressure distribution measurements in a normal population. Gait Posture 2008;27:706–9. 17693087 10.1016/j.gaitpost.2007.07.002

[17] Chodzko-Zajko WJ Proctor DN Fiatarone Singh MA American College of Sports Medicine position stand. Exercise and physical activity for older adults. Med Sci Sports Exercise 2009;41:1510–30. 10.1249/MSS.0b013e3181a0c95c 19516148

[18] Buchan DS Boddy LM Despres JP Utility of the hypertriglyceridemic waist phenotype in the cardiometabolic risk assessment of youth stratified by body mass index. Pediatr Obesity 2016;11:292–8. 10.1111/ijpo.12061 26251875

[19] Herbert P Sculthorpe N Baker JS Validation of a six second cycle test for the determination of peak power output. Res Sports Med 2015;23:115–25. 25720917 10.1080/15438627.2015.1005294

[20] De Vito G Bernardi M Forte R Effects of a low-intensity conditioning programme on (V)over-dot O-2max and maximal instantaneous peak power in elderly women. Eur J Appl Physiol Occup Physiol 1999;80:227–32. 10453925 10.1007/s004210050586

[21] Whyte LJ Gill JM Cathcart AJ Effect of 2 weeks of sprint interval training on health-related outcomes in sedentary overweight/obese men. Metab Clin Exp 2010;59:1421–8. 20153487 10.1016/j.metabol.2010.01.002

[22] Sayers SP Gibson K Effects of high-speed power training on muscle performance and braking speed in older adults. J Aging Res 2012;2012:426278. 22500229 10.1155/2012/426278 PMC3303692

[23] Martin R Buchan DS Baker JS Sprint interval training (SIT) is an effective method to maintain cardiorespiratory fitness (CRF) and glucose homeostasis in Scottish adolescents. Biol Sport 2015;32:307–13. 26681833 10.5604/20831862.1173644 PMC4672162

[24] Tjonna AE Lee SJ Rognmo O Aerobic interval training versus continuous moderate exercise as a treatment for the metabolic syndrome: a pilot study. Circulation 2008;118:346–54. 18606913 10.1161/CIRCULATIONAHA.108.772822 PMC2777731

[25] Tjonna AE Stolen TO Bye A Aerobic interval training reduces cardiovascular risk factors more than a multitreatment approach in overweight adolescents. Clin Sci 2009;116:317–26. 18673303 10.1042/CS20080249

[26] Boudou P Sobngwi E Mauvais-Jarvis F Absence of exercise-induced variations in adiponectin levels despite decreased abdominal adiposity and improved insulin sensitivity in type 2 diabetic men. Eur J Endocrinol 2003;149:421–4. 14585088 10.1530/eje.0.1490421

[27] Boutcher SH High-intensity intermittent exercise and fat loss. J Obesity 2011;2011:868305. 10.1155/2011/868305 PMC2991639 21113312

[28] Gillen JB Percival ME Tarnopolsky MA Low-volume high-intensity interval training reduces abdominal adiposity and increases lean mass in overweight women. Med Sci Sports Exercise 2012;44:237–8.

[29] Gillen JB Percival ME Ludzki A Interval training in the fed or fasted state improves body composition and muscle oxidative capacity in overweight women. Obesity 2013;21:2249–55. 23723099 10.1002/oby.20379

[30] Hayes LD Sculthorpe N Herbert P Six weeks of conditioning exercise increases total, but not free testosterone in lifelong sedentary aging men. Aging Male 2015;18:195–200. 26030347 10.3109/13685538.2015.1046123

[31] Hayes LD Grace FM Sculthorpe N Does chronic exercise attenuate age-related physiological decline in males? Res Sports Med 2013;21:343–54. 24067120 10.1080/15438627.2013.825799

[32] Hayes LD Sculthorpe N Herbert P Resting steroid hormone concentrations in lifetime exercisers and lifetime sedentary males. Aging Male 2015;18:22–6. 25353611 10.3109/13685538.2014.977246

[33] Herbert P Hayes LD Sculthorpe N High-intensity interval training (HIIT) increases insulin-like growth factor-I (IGF-I) in sedentary aging men but not masters’ athletes: an observational study. Aging Male 2016;1–6. 10.1080/13685538.2016.1260108 28042739

[34] Goodpaster BH Park SW Harris TB The loss of skeletal muscle strength, mass, and quality in older adults: the health, aging and body composition study. J Gerontol Ser A Biol Sci Med Sci 2006;61:1059–64. 17077199 10.1093/gerona/61.10.1059

[35] Bean JF Herman S Kiely DK Increased Velocity Exercise Specific to Task (InVEST) training: a pilot study exploring effects on leg power, balance, and mobility in community-dwelling older women. J Am Geriatr Soc 2004;52:799–804. 15086665 10.1111/j.1532-5415.2004.52222.x

[36] Melzer I Benjuya N Kaplanski J Effects of regular walking on postural stability in the elderly. Gerontology 2003;49:240–5. 12792159 10.1159/000070404

[37] Riebe D Franklin BA Thompson PD Updating ACSM's recommendations for exercise preparticipation health screening. Med Sci Sports Exercise 2015;47:2473–9. 10.1249/MSS.0000000000000664 26473759

